# Successful Surgical Management of Chronic Lower Limb Lymphedema: A Case Report

**DOI:** 10.7759/cureus.48621

**Published:** 2023-11-10

**Authors:** Sandeep Reddy Ramala, Suresh Chandak, Meenakshi S Chandak, Souvik Sarkar

**Affiliations:** 1 General Surgery, Jawaharlal Nehru Medical College, Datta Meghe Institute of Higher Education and Research, Wardha, IND; 2 Respiratory Medicine, Jawaharlal Nehru Medical College, Datta Meghe Institute of Higher Education and Research, Wardha, IND

**Keywords:** multidisciplinary care, case report, surgical management, lower limb, chronic, lymphedema

## Abstract

Chronic lower limb lymphedema is a challenging and often debilitating medical condition characterized by the abnormal accumulation of lymphatic fluid in the extremities, leading to persistent swelling and discomfort. While this condition can be caused by various underlying factors, early diagnosis, and appropriate management are crucial for improving the patient's quality of life. This case report presents the successful surgical management of chronic lower limb lymphedema in a 30-year-old male patient who had been grappling with this condition for a decade. The patient's journey from the onset of symptoms, including swelling and difficulty in walking, to the eventual diagnosis and treatment is documented herein. Despite seeking medical care from allopathic and homeopathic sources, the patient's condition continued to deteriorate over the years, underscoring the complexity of chronic lower limb lymphedema and its challenges in clinical management. This case highlights the importance of accurate diagnosis, multidisciplinary evaluation, and a comprehensive surgical approach in addressing the complexities of chronic lower limb lymphedema. It also sheds light on the potential complications that may arise during treatment and the postoperative care required to achieve a favorable outcome. By sharing this case, we aim to contribute to understanding this condition and provide insights into the effective management of chronic lower limb lymphedema.

## Introduction

Chronic lower limb lymphedema is a challenging and often debilitating medical condition that continues to perplex and challenge clinicians worldwide [1]. Characterized by the abnormal and persistent accumulation of lymphatic fluid in the extremities, lymphedema results in chronic swelling, discomfort, and a significant impairment of an individual's quality of life [1]. This case report sheds light on the successful surgical management of chronic lower limb lymphedema in a 30-year-old male patient, serving as an illustrative example of the complexities and intricacies associated with the diagnosis and treatment of this condition.

Lymphedema, a condition that frequently develops insidiously, remains a relatively underdiagnosed and underestimated issue in clinical medicine [2]. The physiological and psychological burden on affected individuals intensifies as it progressively advances. Often overlooked or misdiagnosed, this condition warrants greater attention and understanding [3].

The journey of this patient, detailed in this case report, provides an opportunity to explore the challenges and therapeutic options encountered in addressing chronic lower limb lymphedema. Over the course of a decade, the patient grappled with a growing sense of discomfort, as well as a considerable impediment to his ability to walk. Despite persistent symptoms, the initial medical care sought from private clinics and utilizing allopathic and homeopathic treatments failed to alleviate his suffering. This experience resonates with a broader issue: the lack of widespread awareness and understanding of lymphedema, resulting in delayed diagnoses and, subsequently, protracted suffering for patients [2].

The journey towards diagnosing and treating chronic lower limb lymphedema is often fraught with complexities, demanding a comprehensive and multidisciplinary approach. This case report unveils the significance of early and precise diagnosis as a crucial turning point in a patient's quest for an improved quality of life. The attending physician's recommendation for the patient's admission to the plastic surgery department and the subsequent battery of diagnostic tests, which included blood work, electrocardiography, and radiological imaging via X-rays, represented the first pivotal step in this patient's path toward relief.

The subsequent color Doppler and MRI assessments were fundamental in establishing the definitive diagnosis. The color Doppler study revealed a well-defined collection in the right foot, displaying varying solid and cystic elements, suggesting the presence of a solid cystic mass lesion. The MRI further confirmed this, which portrayed the extent of the subcutaneous edema in the dorsum, medial, lateral, and posterior aspects of the leg. Together, these diagnostic procedures provided the crucial evidence required for the physician to diagnose the patient with right lower limb lymphedema, signaling the start of a therapeutic journey that promised hope and restoration [4].

Surgical intervention emerged as the most appropriate course of action to address the chronic lymphedema in the right lower limb. The complexity of this patient's case necessitated a carefully planned and meticulously executed surgical procedure. The case report delves into the specifics of this surgical journey, encompassing Charles's excision to remove the bulk of lymphedematous swelling, the raising of a skin flap, and the intricate vascularized right submental lymph node transfer to treat the filarial lymphedema [5].

The postoperative period, however, introduced its challenges, including the formation of a wound hematoma and a wound gap that required secondary suturing. Comprehensive postoperative care, including analgesics, antibiotics, antacids, and local applications, was instrumental in the patient's recovery and ultimate success of the procedure [6].

The importance of a multidisciplinary approach, from initial evaluation to diagnosis, surgical intervention, and postoperative care, is a prevailing theme throughout this case report. To enhance understanding and treatment of chronic lower limb lymphedema, this report provides a valuable perspective on the complexities and nuances of managing this condition, offering insights that will contribute to a broader and more effective approach to patient care [7].

## Case presentation

A 30-year-old male sought medical attention at the outpatient department of a tertiary care hospital due to complaints of swelling in his right lower limb and difficulty walking. During the initial history-taking, the patient revealed that the swelling had progressively worsened over the past 10 years. He had previously sought medical care from private clinics and received allopathic and homeopathic treatments.

**Figure 1:**
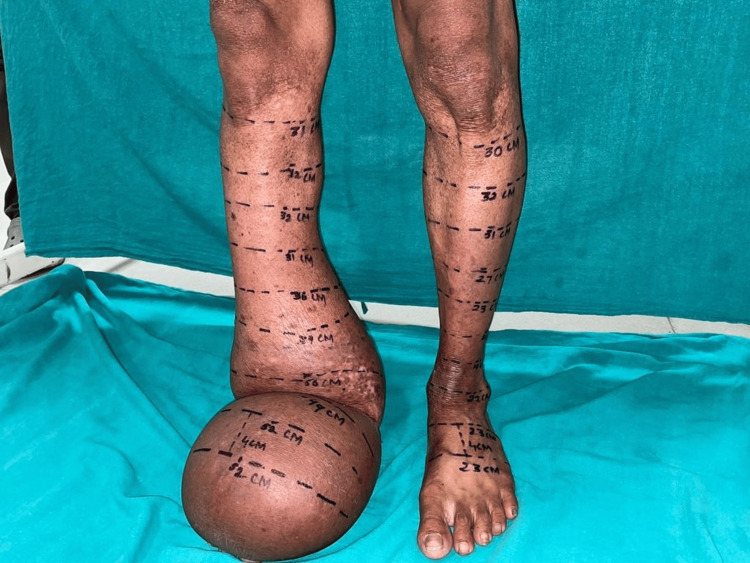
Showing the swelling on the right lower limb, extending from the foot, approximately 20 centimeters proximal to the ankle joint

Upon physical examination, a noticeable swelling was observed in the right lower limb, extending from the foot, approximately 20 centimeters proximal to the ankle joint (Figure 1). The attending physician recommended the patient's admission to the plastic surgery department and suggested a battery of tests, including a blood test, electrocardiogram, and radiological imaging via X-rays. All these tests returned normal results. Consequently, further assessments, such as a color Doppler and MRI, were recommended.

A duplex color Doppler study of the right lower limb, examining both arterial and venous components, 2D B-mode, and color flow, indicated the presence of a well-defined collection in the right foot. This collection exhibited heterogeneity, with some solid cystic elements suggesting the presence of a solid cystic mass lesion.

**Figure 2:**
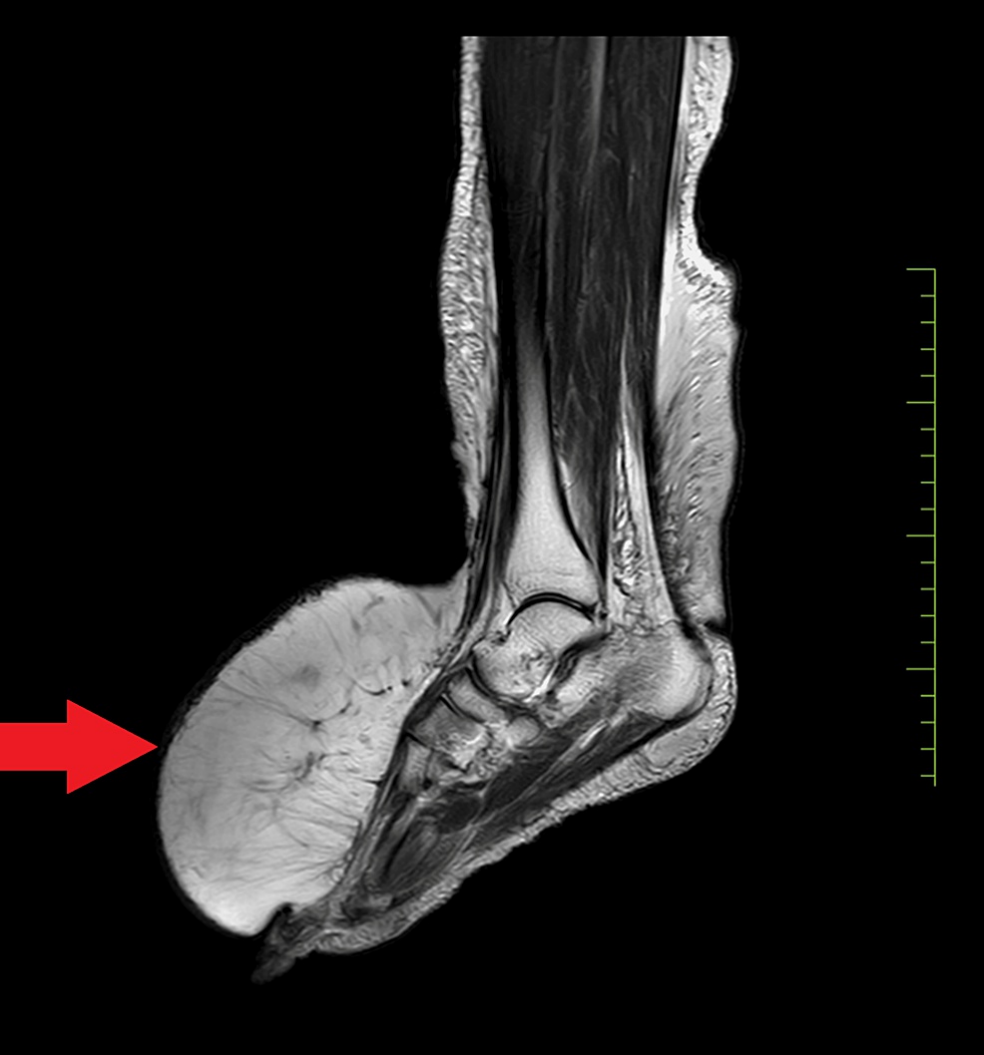
MRI of right leg edema in the dorsum

Subsequently, an MRI of the right leg was conducted, revealing the presence of diffuse subcutaneous edema in the dorsum, medial, and lateral aspects of the foot, as well as the medial, lateral, and posterior aspects of the leg. This edema appeared hyperintense on T2/proton density weighted spectral attenuated inversion recovery (PDW SPAIR) and hypointense on T1 (Figure 2). Following these findings, the physician diagnosed the patient with right lower limb lymphedema and recommended surgical intervention.

**Figure 3:**
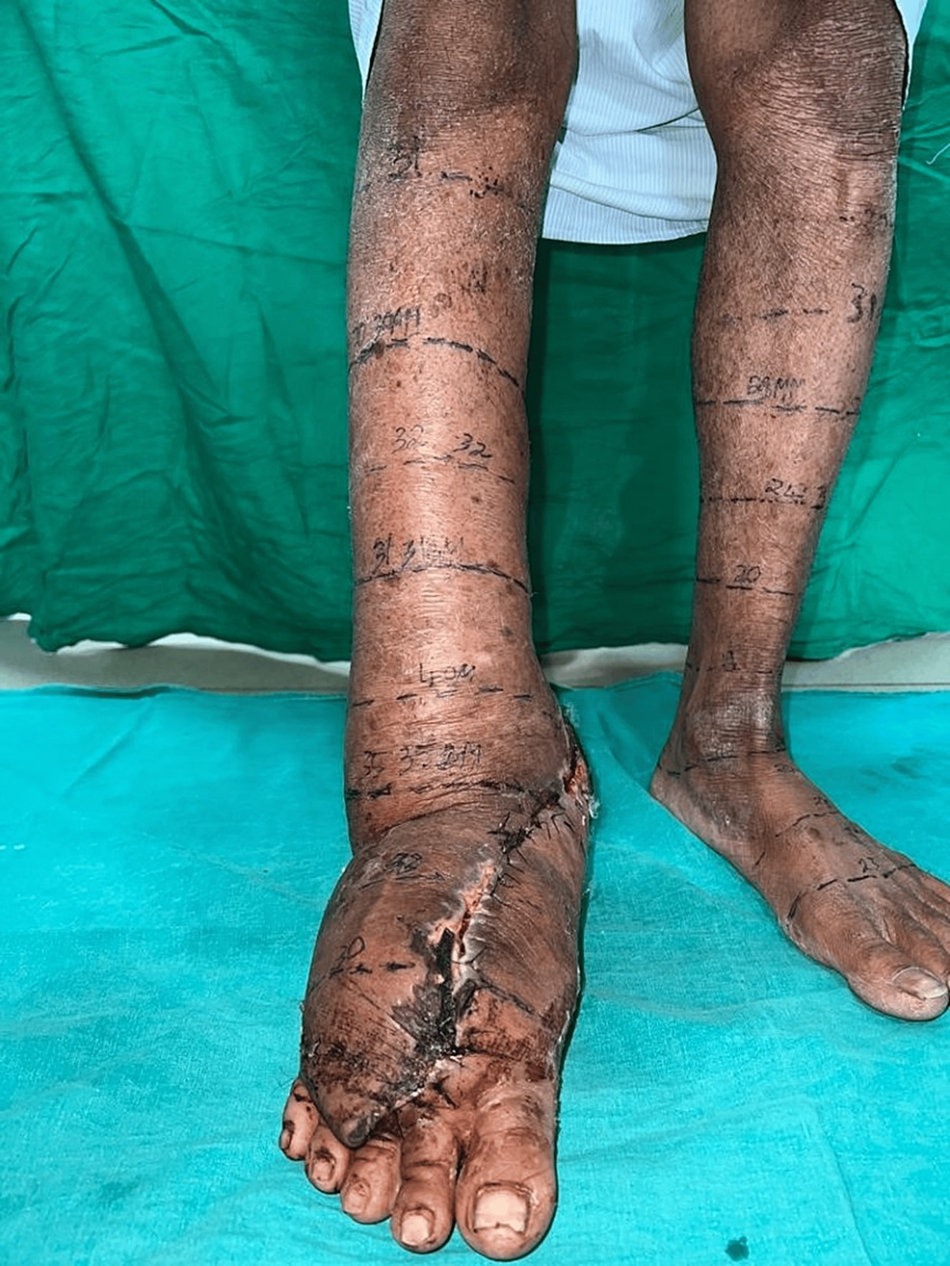
Showing the right leg after the removal of lymphedematous swelling

After thorough counseling and obtaining consent from the patient and their relatives, surgery was performed under general anesthesia. The procedure included Charles's excision to remove most of the lymphedematous swelling after raising the skin flap and a vascularized right submental lymph node transfer to treat right lower limb filarial lymphedema. The flap dissection was performed, and the neck was meticulously closed in layers with a drain. The leg dressing was opened, and hemostasis was successfully achieved, as indicated by the bright red color of the flap bleed (Figure 3).

**Figure 4:**
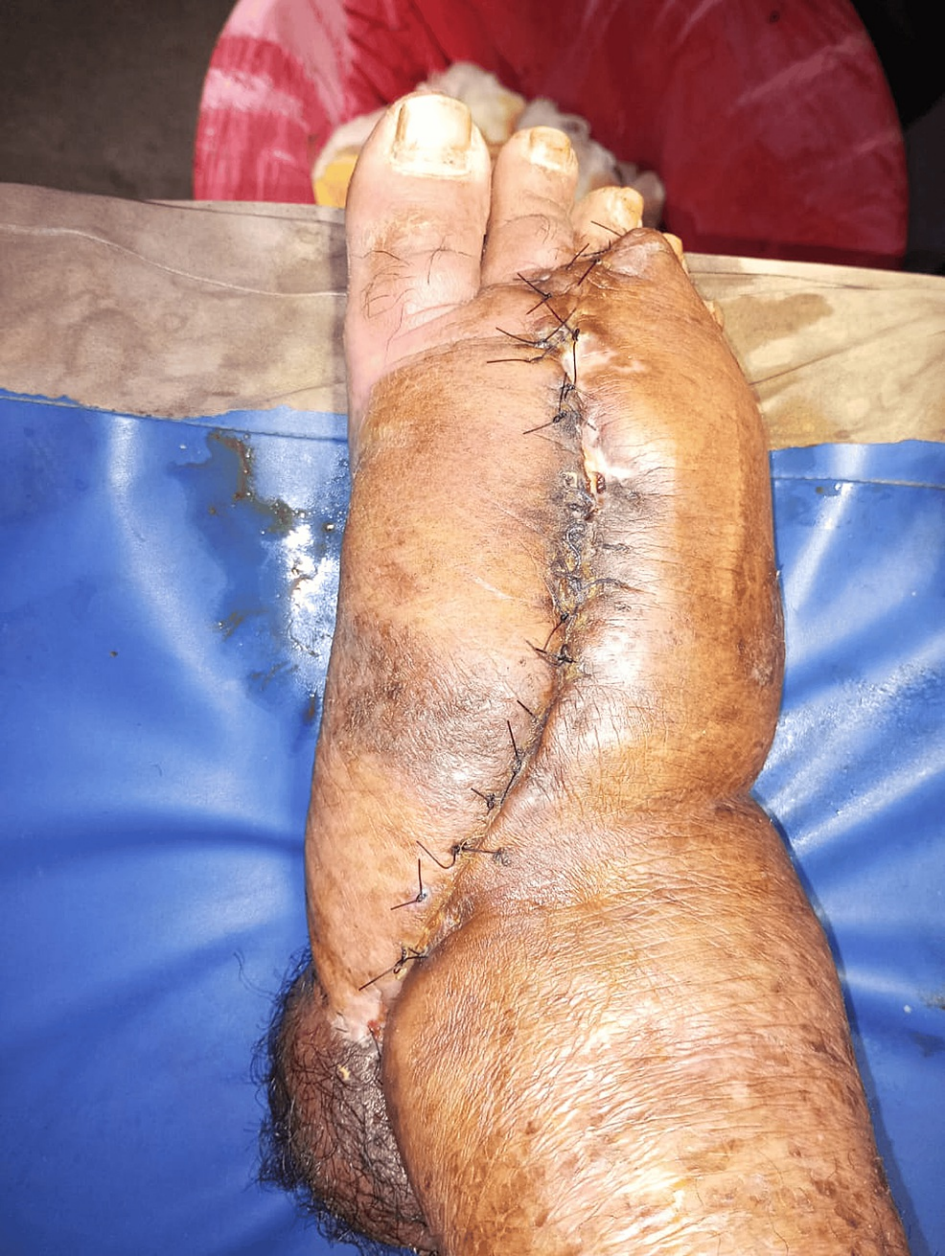
Follow-up image of the patient after one month

Postoperatively, a wound hematoma developed and required drainage through regular washes. A wound gap also formed, necessitating secondary suturing. The patient was managed with analgesics, antibiotics, antacids, and local applications. Following appropriate care, the patient was discharged with multi-layered bandaging and given instructions for regular dressing with Hydroheal (Figure 4). Additionally, the patient has been prescribed Penidure injections (1.2 million units) monthly for one year and doxycycline (100 mg) every 12 hours for a month. The patient was advised to follow up at the plastic surgery outpatient department after one month and instructed to visit the emergency department in case of bleeding, discharge, foul odor, or pain.

## Discussion

Chronic lower limb lymphedema is a challenging condition characterized by the chronic accumulation of lymphatic fluid in the lower extremities. This case report underscores the complexities associated with diagnosing and managing this condition, especially when it has progressed over a prolonged period. The presented case highlights the importance of early diagnosis, a multidisciplinary approach, meticulous surgical intervention, and potential challenges and complications that can arise during treatment.

Early diagnosis of chronic lower limb lymphedema is crucial for a favorable outcome. In this case, the patient's decade-long struggle with swelling and discomfort emphasizes the need for greater awareness and timely identification of lymphedema. As several studies have indicated, delayed diagnosis often results in the condition's progression, leading to more severe symptoms and complications [8,9]. The comprehensive management of chronic lower limb lymphedema necessitates a multidisciplinary approach. This case highlights the pivotal role played by plastic surgeons, radiologists, and other healthcare professionals in evaluating the patient's condition accurately and devising an effective treatment plan. Collaborative care is essential for providing patients with the best possible outcomes [10].

Accurate diagnosis is the cornerstone of successful treatment. In this case, diagnostic tools such as color Doppler and MRI played a vital role in confirming the presence of lymphedema and assessing its extent. These imaging techniques are instrumental in characterizing the condition and guiding treatment decisions [11]. Surgical management is often the most appropriate for chronic lower limb lymphedema, especially when conservative treatments have proven ineffective. The surgical procedures employed in this case, including Charles's excision and lymph node transfer, reflect the complexity and individualized nature of treating lymphedema. Successful surgical management can significantly improve a patient's quality of life [12].

Postoperative care is equally critical to the overall success of lymphedema treatment. Complications, such as wound hematomas and the formation of wound gaps, as seen in this case, are not uncommon. These issues require prompt attention and appropriate interventions. This report highlights the need for vigilant postoperative care, including meticulous wound management and pharmacological support [13]. This case emphasizes the importance of long-term follow-up and patient education. The provision of instructions for regular dressing, prescribed medications, and scheduled follow-up appointments is essential for ensuring the continued success of lymphedema treatment. Moreover, patient education plays a pivotal role in fostering self-management and prevention of future complications [14].

## Conclusions

In conclusion, the case report detailing the successful surgical management of chronic lower limb lymphedema emphasizes the critical components necessary for effectively addressing this challenging condition. Early diagnosis stands out as a pivotal factor, given the potential for delayed diagnosis to result in the progression of lymphedema and its associated complications. This underscores the need for heightened awareness among healthcare professionals and the general public regarding lymphedema's early signs and symptoms. Furthermore, a multidisciplinary approach involving collaboration between various medical specialists is essential for a comprehensive evaluation and management of lymphedema. The complex nature of this condition necessitates a collaborative effort to ensure the best possible outcomes for patients.
